# Bibliographical Mapping of Research into the Relationship between In Ovo Injection Practice and Hatchability in Poultry

**DOI:** 10.3390/vetsci10040296

**Published:** 2023-04-17

**Authors:** Gabriel da Silva Oliveira, Concepta McManus, Cristiane Batista Salgado, Vinícius Machado dos Santos

**Affiliations:** 1 Faculty of Agronomy and Veterinary Medicine, University of Brasília, Brasília 70910-900, Brazil; gabriels.unb@gmail.com (G.d.S.O.);; 2 Laboratory of Geosciences and Human Sciences, Federal Institute of Brasília—Campus Brasília, Brasília 70830-450, Brazil; 3 Laboratory of Poultry Science, Federal Institute of Brasília—Campus Planaltina, Brasília 73380-900, Brazil

**Keywords:** embryos, feeding in ovo, hatching eggs, poultry production, VOSviewer

## Abstract

**Simple Summary:**

Poultry mortality in the embryonic stage makes it difficult to achieve one of the main objectives of the poultry sector, i.e., constant increase in productivity. If well executed with safe and efficient substances, the in ovo injection technique can be an intervention alternative to enhance the development of more resistant, healthy, quality birds, with substantial positive effects on poultry production. For the commercial implementation of any intervention, information from the vast literature must be compiled in order to understand its effects on production parameters. Thus, this review aims to assess how injecting different substances in ovo influences hatching results, including the reported effects on embryo and chick health parameters. Bibliographic mappings of co-authorship of citations, co-occurrence of keywords and bibliographic coupling were also performed for articles based on the in ovo injection technique and hatchability.

**Abstract:**

Recent advances in poultry practice have produced new tools enabling the poultry industry to increase productivity. Aiming at increasing production quality, varying protocols of in ovo injection facilitate the introduction of exogenous substances into the egg to complement the nutrients that support embryonic development up to hatching, which are already available in the internal and external compartments. Due to embryonic sensitivity, adding any substance into the egg can be either advantageous or disadvantageous for embryonic survival and can influence hatch rates. Thus, understanding the relationship between poultry practices and production rates is the first step towards successful commercial application. This review aims to assess the influence on hatch rates of injecting different substances in ovo, including effects on embryo and chick health parameters where these are reported. Bibliographic mappings of co-authorship of citations, co-occurrence of keywords, and bibliographic coupling based on the in ovo injection technique and hatchability parameters were also performed. Using the Scopus database, 242 papers were retrieved, reviewed, and submitted for bibliographic mapping using the VOSviewer^®^ software. This review provides a broad overview of just over 38 years’ research on the subject, revealing that studies have significantly increased and peaked in 2020, being produced primarily by US researchers and published primarily in the journal *Poultry Science*. It also reveals that despite negative reports relating to some substances in the embryo, in ovo delivery of substances may possibly change the poultry industry for the better in terms of production rates (hatchability) and/or poultry health.

## 1. Introduction

Farmers collaborate with poultry scientists to achieve maximum production capacity and quality. Health is one of the foundations for sustaining productivity in the poultry industry and ensuring human safety. The guarantee of health requires continuous work with broad, diversified, and flexible solutions. These solutions must be protective or enhancing in nature, limiting damage to health and welfare, and/or improving poultry’s physical and physiological qualities.

Hatchability designates the percentage of chicks hatched alive from a given sample of eggs. The hatchability rate is used worldwide by the poultry industry to monitor the productivity of each hatchery. The objective is to maintain this rate close to 100%. The greater the hatchability, the greater the guarantee that everything proceeded correctly throughout embryonic development. However, this is not a rule. Chicks that hatch with imperceptible or perceptible deformations can be counted in hatchability rates and are usually discarded before going to the farm, or may go to the farm but die shortly afterwards. Therefore, health and quality parameters must be analyzed along with hatchability to ensure high production and quality before and after hatching.

This parameter is decisive in the advance or decline of poultry production, with significant economic effects. With the hatchability value, the hatchery can predict not only its own economic return, but also that of the production unit, considering the expected mortality rate at the hatch–slaughter interface. In addition, hatchability can serve as a solid reference to monitor and evaluate experiments performed directly on eggs, which can influence embryonic survival and support the identification of product deficiencies and solutions to overcome these. According to Gonzalez et al. [1], an improvement in hatchability percentages can be obtained by altering factors inherent to the eggs. Examples of factors include the sanitization of hatching eggs [2,3,4] and the physical parameters of incubation [5]. Another factor is in ovo injection [6].

**Figure 1:**
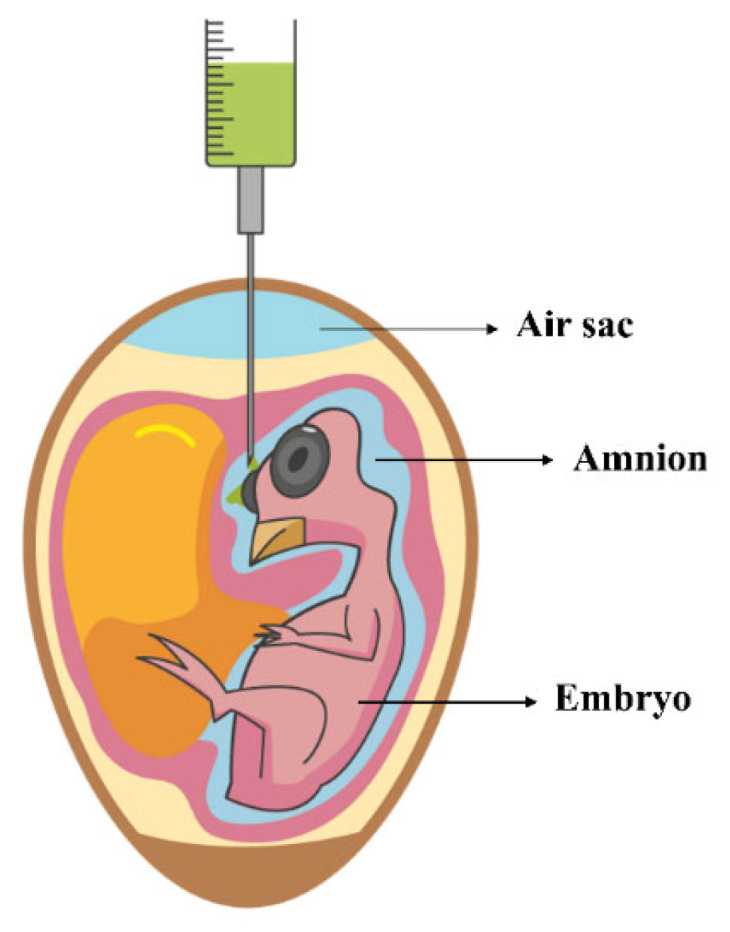
Delivery of substances into the amniotic fluid during embryonic development.

Avian health before and after hatching depends on their exposure to egg nutrients, which include vitamins, minerals, lipids, carbohydrates, and proteins [7,8]. The balance between the nutritional status of the egg and the short developmental window of the embryo is essential for the complex delivery of nutrients to the embryo in a limited time. Nutritional insufficiency during embryo formation, which may be associated with genetic factors, can disrupt the progression of healthy and viable development [9]. This is likely to harm the bird’s health and survival throughout its life. An insufficient nutritional load for the embryo imposes the need for nutritional complementation to supply all the embryonic demands until hatching. This is carried out through early or late injection of exogenous nutrients into the egg during embryonic development [8,10]. The main point of deposition or application of nutrients is the amnion (Figure 1) [8]. This process influences embryonic physiology, reflected in hatchability rates [11].

In ovo injection is a practical solution that is revolutionizing poultry production due to its immediate beneficial effects during embryogenesis or post-hatch. Nevertheless, some harmful effects have been seen. While Subramaniyan et al. [12] reported that in ovo injection of L-arginine (100 µg/µL/egg) benefits embryo survival rate, hatchability, and chick weight, Araújo et al. [13] observed that in ovo injection of a product containing canthaxanthin at different concentrations (0.35 to 0.65 mg/mL/egg) impaired hatchability and chicks’ physical quality.

In the present work, we reviewed published studies to determine whether the benefits in terms of hatchability outweigh the harm of injecting different substances in ovo, in relation to the influence of intrinsic or extrinsic factors. Bibliographic mappings of co-authorship of citations, co-occurrence of keywords, and bibliographic coupling relating to in ovo injection and hatchability parameters were also performed.

## 2. Bibliographic Mapping

### 2.1. Material and Methods

The process of obtaining bibliographic information related to the in ovo injection technique and its effects on hatchability was divided into several steps (Table 1).

Scopus was used as a source of access to bibliographic data, as it is a reliable global database that stores important scientific data, including data relating to poultry sciences. The search keywords were defined based on the research objective. The number of research papers retrieved from Scopus and eligible for bibliometric analysis comprised 242 documents, covering just over 38 years. Inclusion criteria were research papers written and published in English, while exclusion criteria were all documents that did not meet the inclusion criteria (reviews, conference papers, and research articles written and published in other languages). The database was extracted from Scopus in CSV Microsoft Excel format and processed with VOSviewer^®^ software to generate connection/relationship networks between the authors, countries, and scientific journal sources of the published studies. VOSviewer^®^ was used because it is a practical and easy-to-use software package with good graphic quality, which allows the analysis of metadata from different databases and has excellent integration and communication with the SCOPUS database [14].

### 2.2. Results

**Figure 2:**
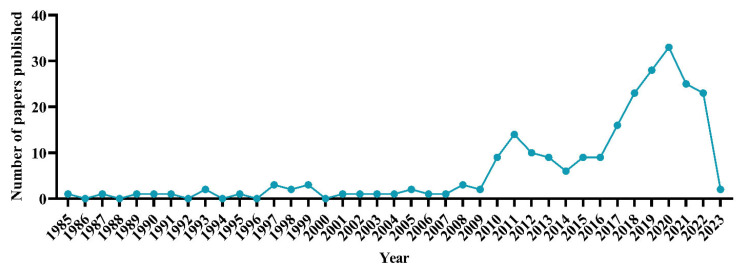
Number of publications from 1985–2023 related to the in ovo injection technique with a focus on hatchability.

The 242 papers on the in ovo injection technique and its effects on hatchability, selected and analyzed from 1 January 1985 to 14 January 2023, are shown in Figure 2, highlighting the annual numbers of publications. The oldest paper was written by Gildersleeve et al. [15] on the effects of injecting diethylstilbestrol in ovo, and was published in *Teratology*. Comparing the decades 1991–2001 (5.31% of total publications), 2002–2012 (18.37%), and 2013–2023 (74.69%; to 14 January 2023), it can be observed that from the first decade to the second, the numbers of published articles increased 3.5 times, from the first decade to the third 14.1 times, and from the second to the third the numbers increased 4.1 times. The number of publications on the topic increased from 10 in 2011, reaching a peak (n = 33 publications) in 2020. These findings show that the field of research has started to gain more recognition in recent years and that the quantity of research prior to 2010 was highly limited.

**Figure 3:**
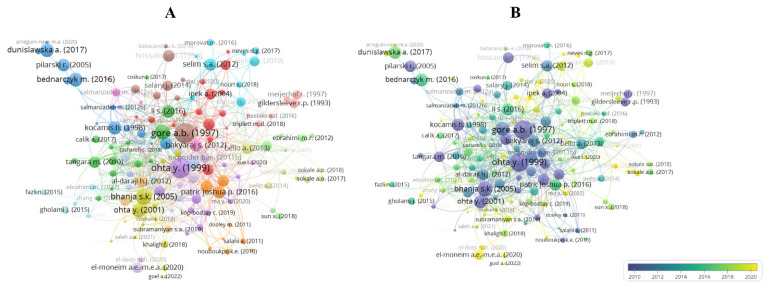
Citation-based document network.

A paper with many citations is positive feedback for the group of researchers that developed it, demonstrating that it represents high-quality, relevant, and impactful work within the research area. It should be noted that it is not a rule that older studies are more frequently cited than more recent ones. Based on data obtained from Scopus, 167 publications had at least three citations. The authors of the respective publications are listed in Figure 3A. Gore and Qureshi [16], Ohta et al. [17], and Bhanja and Mandal [18] have the most cited papers, comprising the top three internationally (Table 2). Those papers focus their analysis mainly on the impact of administration of substances in ovo on embryonic development, hatchability, immune health of birds, their digestive system, and post-hatch performance, proving to have a high level of influence on subsequent published research. Gore and Qureshi [16] studied the beneficial relationship of in ovo exposure to vitamin E and the immune system of turkeys and broilers. Ohta et al. [17] explored amino acid supplementation in ovo and its relationship with embryonic growth, hatchability, and chick weight. In one of their conclusions, the authors reported that amino acids injected into the yolk sac may be an effective means of increasing chick size without decreasing hatchability. Bhanja and Mandal [18] aimed to standardize the in ovo injection of amino acids and evaluate their effects on pre- and post-hatching performance, immune response, and development of digestive organs. Some of their findings showed that amino acids did not influence hatchability and digestive organ weights at 21 days, and that amino acids can act as immunomodulators. Figure 3B is a representation of the most frequently cited documents over the study period. The most recent study among the 10 most cited, ranking seventh with 57 citations, aimed to design synbiotics in vitro and validate them in broilers after in ovo delivery [19]. This confirms our statement above, showing that more recent studies may have a more significant contribution to the field than older ones, although they may not yet have been cited.

**Figure 4:**
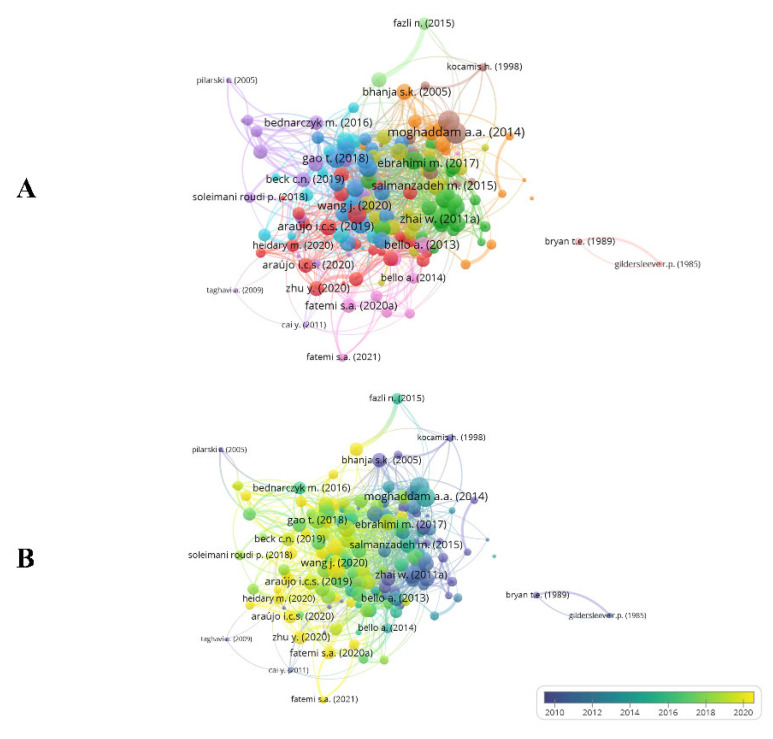
Bibliographic coupling of documents.

Bibliographic coupling connects two papers that have another paper in common [26]. This review carried out bibliographic coupling between documents with at least three citations, resulting in 167 documents forming 11 clusters (Figure 4A,B). Based on total link strength, the papers by Moghaddam et al. [27], Moghaddam et al. [28], and Zhai et al. [29] were found to have the highest bibliographic coupling forces (total link strength of 48.00, 47.00, and 37.00, respectively). Clusters were formed by studies with very similar objectives. Our findings also show that the most frequently cited papers (Table 2) are not always those with the greatest bibliographic coupling strength.

**Figure 5:**
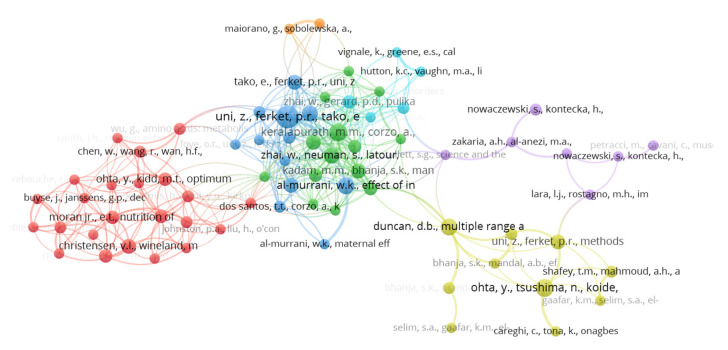
Reference co-citation network.

References with at least three citations were selected for co-citation analysis. In the analysis, seven clusters were formed (Figure 5) and the most frequently cited reference (present in the blue cluster) was “In ovo feeding improves the energy status of late-term chicken embryos” by Uni et al. [30] published in *Poultry Science*, with 14 co-citations and a total link strength of 94. In that paper, researchers measured the effects of in ovo feeding with highly digestible carbohydrates and β-hydroxy-β-methylbutyrate.

**Figure 6:**
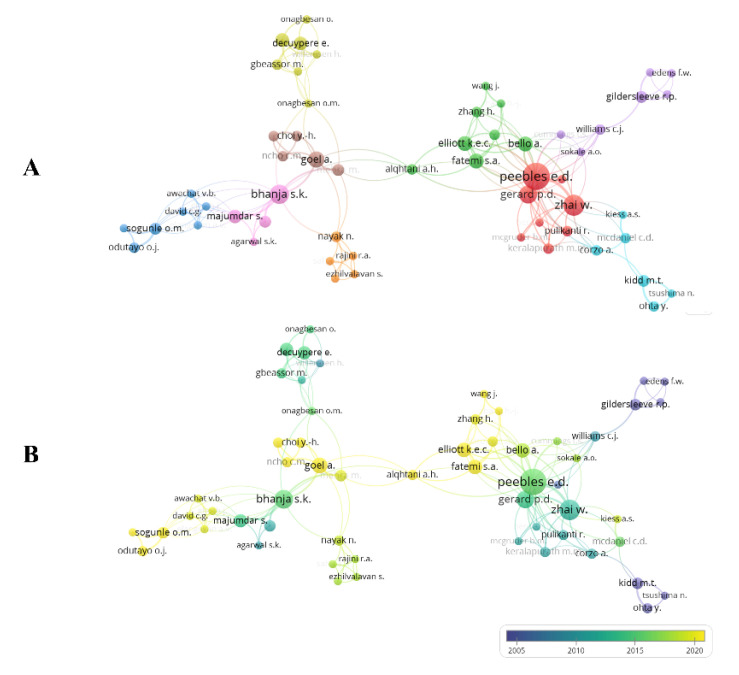
Authors network based on number of documents.

Of 914 authors, 182 had at least two documents, but only 57 with links. The authors were grouped (represented by the colors of lines and circles) into nine clusters (Figure 6A). Researchers Peebles (20 publications), Zhai (12 publications), and Bhanja (10 publications) were the authors with the most publications, showing intense connectivity and collaboration with other researchers within the circuits where they are present. However, there was no connectivity from Peebles and Zhai to Bhanja. Clusters were formed among authors who had strong bonds. At least one author from each group contributed to another group. Zhai is the oldest contributor in the field, compared with Peebles and Bhanja (Figure 6B). Korean (e.g., Goel), Nigerian (e.g., Odutayo), and US (e.g., Fatemi) researchers are among the most recent contributing researchers (Figure 6B).

**Figure 7:**
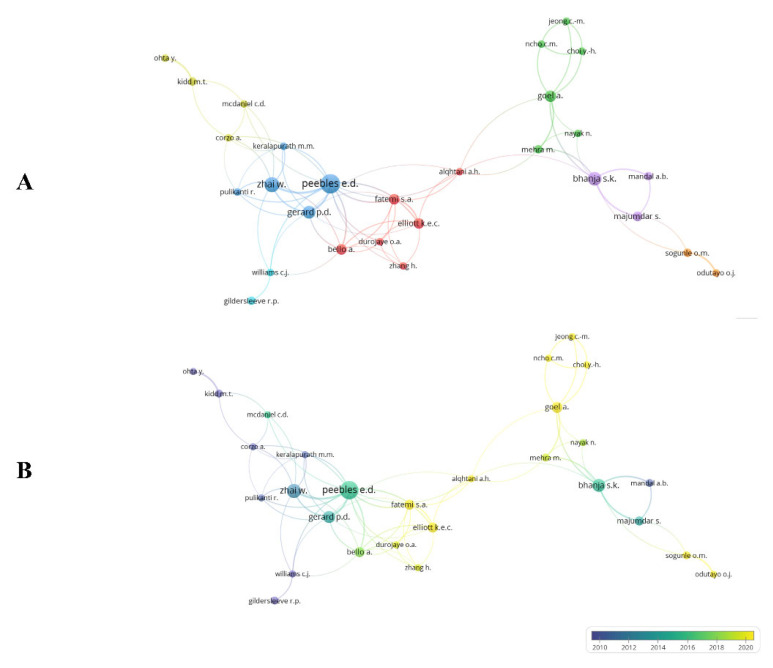
Network of authors based on numbers of citations.

A total of 914 authors were identified. By limiting the numbers to four citations and four documents per author, 28 authors were included in the citation network (Figure 7A). Highest ranked were Peebles (415 citations, 19 links), Zhai (352 citations, 21 links), and Kidd (230 citations; 21 links). It was observed that the author with the most publications did not always have the highest number of citations, as was the case for Bhanja [more publications (10) and fewer citations (208)] and Kidd (four publications and 230 citations). Considering the entirety of the time when the research was developed (1985–2023), Peebles can be considered the most influential researcher in the field, and his number of citations reflects the quality and relevance of his research. He was most cited around the year 2016 (Figure 7B), when the number of publications began to increase before reaching a peak (Figure 2).

**Figure 8:**
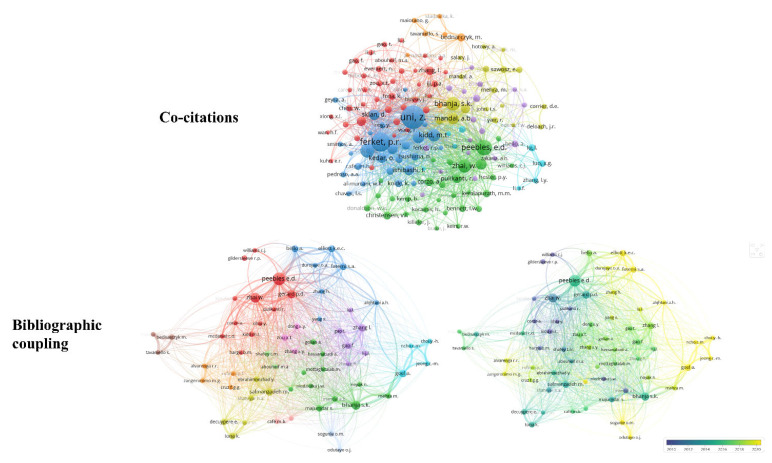
Network of co-citation and bibliographic coupling of authors.

Authors who had at least 20 citations from 242 publications underwent co-citation analysis. Based on the results of the analysis, the authors were grouped into seven clusters (Figure 8); the most frequently co-cited are Uni (573 citations), Ferket (376 citations), and Peebles (260 citations). These are not independent researchers. Comparing the clusters, despite these three authors collaborating with each other, it can be seen that Peebles (green cluster nucleus) is part of a different network of researchers than Uni and Ferket (blue cluster nucleus). Peebles, Zhai, and Gerard are the researchers with the highest bibliographic coupling strength (total link strength 8166, 5944, 4925, respectively). This can be justified by the fact that these researchers published several studies together; the network of authors shows these authors strongly connected (Figure 8), which means they each have a highly similar number of references.

**Figure 9:**
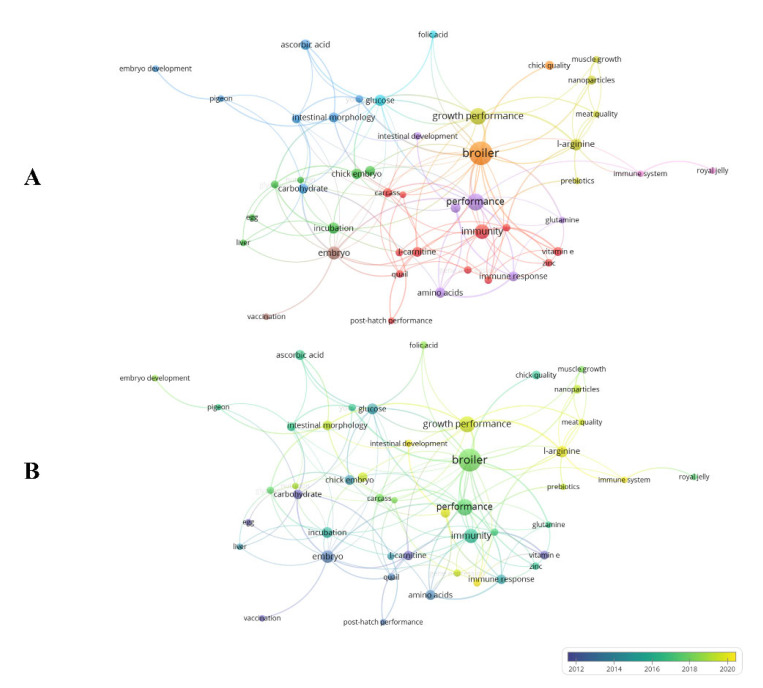
Co-occurrence network of authors’ keywords.

All keywords with at least three occurrences were selected. Of a total of 558 keywords co-occurrence, 70 meet the threshold, 46 with links. Keywords used in the search have been removed. Keywords with the same meaning were merged. Broiler (36 occurrences), performance and growth performance (18 and 17 occurrences, in this sequence = 35 occurrences), and immunity (14 occurrences) were the most prominent terms, showing strong correlation. They present the largest circles and are close together (Figure 9A). These data show that broiler breeder eggs were the main materials used in studies in this area, and that the in ovo injection technique is one of the current trends to improve broiler performance, as well as hatchability and the immunity of the poultry. In general, the nine groupings formed involve the products tested in the in ovo application, their effects, and possible targets in the physiology of the birds. Figure 9B demonstrates the evolutionary process of the keywords by the color gradient from blue to yellow. The keyword co-occurrence network that included the most prominent keywords began to gain more importance only after 2018, showing that the use of the in ovo injection technique in broiler breeder eggs remains recent.

**Figure 10:**
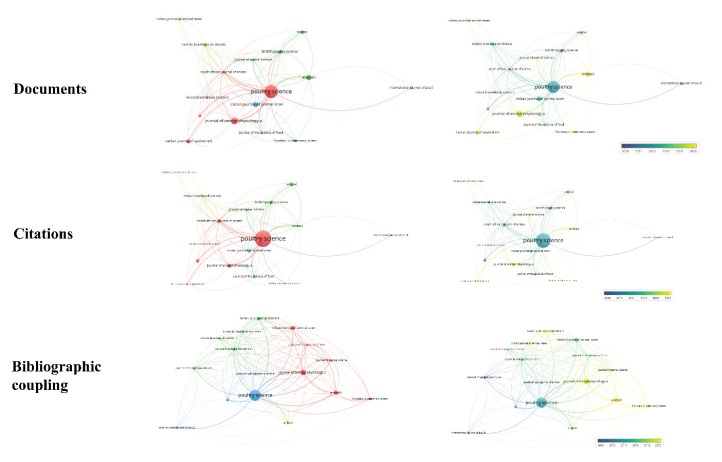
Network of journals based on numbers of documents, citations, and bibliographic coupling.

The 242 reviewed papers were published in 96 different journals. Journals with a minimum of three citations and three publications were viewed in VOSviewer^®^ to identify which journals globally publish and disseminate the most research on the in ovo injection technique with an emphasis on hatchability. Only 16 met this condition, as shown in Figure 10. Subsequently, the 10 journals that published the most studies were included in Table 3. In addition to receiving the most papers for publication, *Poultry Science* (impact factor 4.014) is the most cited journal and has the greatest bibliographic coupling strength (based on total link strength = 372.07) (Figure 10). This journal is the most internationally relevant (based on impact factor) dedicated to the publishing of poultry research. Other world-renowned journals that published large numbers of papers or had many citations in the area of in ovo injection are the *Journal of Animal Physiology and Animal Nutrition* (impact factor 2.718), which publishes papers in areas such as animal physiology, animal nutrition, and feeding technology, *Animals* (impact factor 3.231), which publishes papers in any field of study involving animals, and *Animal* (impact factor 3.73), which publishes cutting-edge papers related to animals (farmed or managed). *Animals* was the journal that published the most articles on the subject and has recently been the most cited (Figure 10).

**Figure 11:**
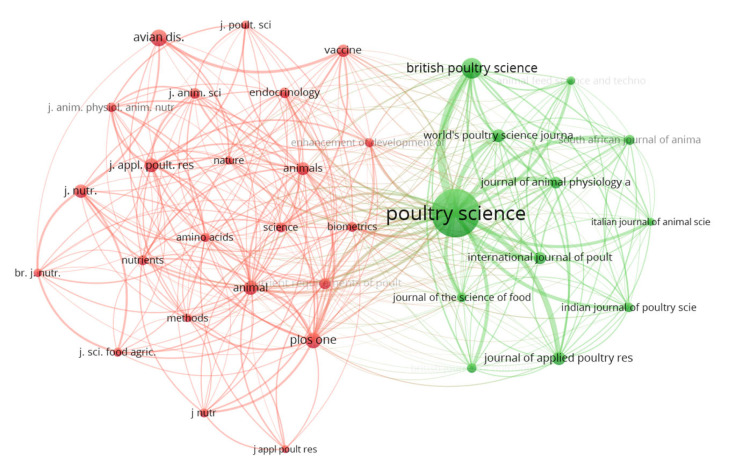
Journal co-citation network.

Journals with at least 23 citations were included in the co-citation analysis. Of the 3560 total citations, 34 journals met this criterion. Two large groups were formed (Figure 11), and the ranking is led by *Poultry Science* (2045 citations). Once again it demonstrated its capacity for contributing, sharing, and disseminating high quality work. In this regard, it appears that *Poultry Science*, as a specific and relevant poultry journal, is of most interest to researchers in this field.

**Figure 12:**
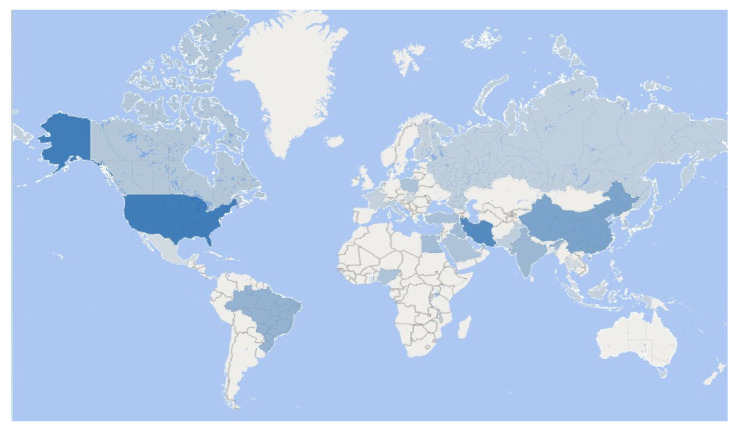
Heat map for numbers of papers by country.

**Figure 13:**
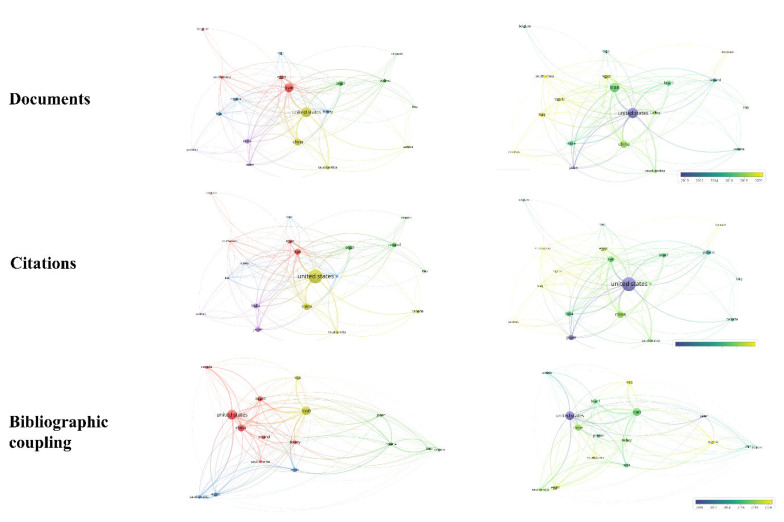
Network of countries based on numbers of documents, citations, and bibliographic coupling.

The publications were prepared by researchers from 33 countries (Figure 12). To analyze the number of publications per country, a limit of at least five documents per country was established, totaling a network of 19 countries (Figure 13). Authors’ countries were grouped into four clusters. USA appears as the most productive (57 documents), followed by Iran (48 documents) and China (32 documents). As the leading countries, USA is partnered primarily with Iran and China. These three countries published more than half (56.61%) of the total number of publications on the subject, demonstrating mastery, productivity, activeness, and dedication to the development of this new area of research. However, the US mainly contributed to the research field around 2010, while Iran did so over 6 years later (around 2016) and China over 8 years later (around 2018) (Figure 13). Korea, Nigeria, and Iraq are the countries that have recently contributed the most to the field (Figure 13). USA and Iran are the countries that collaborate most with others (15 links). The citation ranking leaders, considering a minimum of five citations per country, are composed of the same countries supported by the largest nodes in the country network (Figure 13), in the following order: USA (1384 citations), China (415 citations), and Iran (353 citations). According to the bibliographic coupling analysis (Figure 13), these countries also had the highest bibliographic coupling strength (total link strength of 903.51, 681.96, and 826.17, respectively).

## 3. Effects of In Ovo Injection Practice on Hatchability

To evaluate the effects of in ovo injection on hatchability, studies were selected (n = 86) from the past two decades (2002–2022) that included hatchability (keyword used as a filter to aid in the selection of papers) as an evaluative parameter of substances applied in ovo (Table 4). Improving hatchability is important for maintaining the quality of the poultry chain. Under improved hatchability conditions, embryonic formation, growth, health, and immunity should be minimally affected. Positive effects were reported in 17.44% of the reviewed studies (Table 4). Among the substances tested in these studies were L-arginine, coenzyme Q10, and honey. In contrast, protocols for the in ovo application of harmful substances to poultry were reported in 13.95% of published articles (Table 4). Among the substances tested in those studies were basil seeds, nano zinc oxide, and creatine monohydrate. In addition to high productivity, poultry must be physically and physiologically healthy. Therefore, neutral effects on hatchability are favorable for the poultry industry only if accompanied by improvements in the development and health of the poultry. Various injected substances including creatine pyruvate, betaine hydrochloride, and silver nanoparticles had no effect on hatchability, as demonstrated by 44.19% of the reviewed studies. Results combining positive, negative, or non-existent effects on hatchability for the same substances injected in ovo (e.g., threonine, royal jelly, and zinc) were reported in 32.56% of the studies. The results of hatchability after injection of substances in ovo can be explained or accompanied by hypothetical or observed effects on embryos and poultry in the early stages after hatching (Table 5).

Based on the studies reviewed (Table 4), solution concentration and volume during in ovo injection practice are perhaps the most important factors influencing hatchability, regardless of the substance used. Up to slaughter, poultry goes through a period of morphological, physiological, and nutritional transition, being able to undergo manipulations in the external-to-internal direction while still in the embryonic stage. These manipulations include injecting substances into the egg, aiming to provide poultry with an improved viable life cycle in terms of development, hatching, growth, and resistance to adverse events [8]. However, depending on factors such as concentration and volume of solution, the expected benefits of in ovo injection can be converted into harm (e.g., interruption of the poultry’s life before hatching), or in some situations may have no effect (Table 4 and Table 5).

The concentration and volume determine the effects of the injected substance on avian survival, directly influencing the hatchability rate. Thus, these are important factors for determining conflicting hatch rates. Gao et al. [50] demonstrated that eggs injected with 2% L-arginine at 17.5 days of incubation hatched significantly less than eggs injected with the same substance at 0.5 and 1%, those injected with 0.75% physiological saline solution, and those not injected. They explained this by reporting that the highest concentration tested (2%) is probably toxic to the embryo and can unbalance the internal content of amino acids. Nŉanle et al. [52] reported that eggs injected with 0.5 μg/mL of Moringa oleifera leaf extract at 18 days of incubation hatched significantly more than eggs injected with the same extract at 5 or 50 μg/mL or not injected. Higher hatchability at the lowest extract concentration was associated with adequate feed transit and nutritional absorption, which increased lipid metabolism, resulting in greater energy production for hatching. Mahjar and Al-Salhie [108] reported that eggs injected with 150 μL of alcoholic garlic extract (5 g/50 mL) on day zero of incubation hatched similarly to eggs injected with 50 or 100 μL of the same extract and significantly more than eggs injected with 100 μL distilled water or not injected. Their analysis indicated that 50 or 100 μL of extract had no significant effect on hatchability. According to these authors, better hatchability at the extract highest volume may be associated with the antimicrobial and antioxidant qualities of garlic constituents, with effects limited to newborn chicks.

Researchers need to redesign protocols for injecting substances in ovo that have adverse side effects due to the concentration and volume of the solution. Prior knowledge of the substance’s toxicity is necessary to achieve the optimal concentration and volume more quickly, or to discard the substance immediately if it is toxic at the minimum effective concentration. This review shows that the practice of in ovo injection performed in either the prenatal or perinatal stages of embryonic development, using safe and efficient products, can improve hatchability. However, in most cases, despite improving the health parameters of the poultry, the products were not efficient enough to influence hatchability. In a minority of cases, worsening of hatchability was reported. These results are largely dependent on the intrinsic factors of the practice. Because the products are injected directly into the embryonic environment, the health parameters of poultry appear always to undergo positive or negative changes, even if these are minimal or not significant. The positive effects stand out and many are similar between the different products tested, suggesting that they act on deficient targets common in avian organisms.

## 4. Conclusions

This review provides a broad overview of just over 38 years’ research on the subject, revealing that studies significantly increased and peaked in 2020, were produced primarily by US researchers, and were published primarily in the renowned journal *Poultry Science*. It can be seen that in the last 12 years, research on the subject has taken a great step forward due to the interest that the poultry sector has in practices that can strengthen and leverage its production systems. It is hoped that this review will help researchers pave the way for establishing and validating protocols that are feasible for official inclusion in commercial poultry practices. The difficulty in standardizing between studies the stages of the in ovo application protocol (regardless of the substance applied) is a barrier that can be overcome with the development of field research supervised by a collaborative network involving different countries, researchers, research centers, and the poultry sector.

This review also reveals that despite some negative reports relating to substances in the embryo, in ovo delivery of substances has the potential to change the poultry industry for the better in terms of production rates (hatchability) and/or poultry health. To make this technology applicable to the poultry industry there is a demand for active, efficient, safe, reliable, and cheap substances. These need to be tested at laboratory and industrial levels. Future studies must focus on elucidating the main embryonic physiological targets of substances delivered in ovo that are most likely to improve hatchability.

## Figures and Tables

**Table 1:** Bibliographic information search protocol.

Data Base	Scopus
Research selection points	Title, abstract, keywords
Keywords	Hatchability, in ovo injection, in ovo feeding, in ovo nutrition, and in ovo delivery
Search mode	(TITLE-ABS-KEY (hatchability) AND TITLE-ABS-KEY (in AND ovo AND injection) OR TITLE-ABS-KEY (in AND ovo AND feeding) OR TITLE-ABS-KEY (in AND ovo AND nutrition) OR TITLE-ABS-KEY (in AND ovo AND delivery)) AND (LIMIT-TO (PUBSTAGE, “final”)) AND (LIMIT-TO (DOCTYPE, “ar”)) AND (LIMIT-TO (LANGUAGE, “English”))
Publication coverage period	Until 14 January 2023
Data extracted from the database	Title and abstract of the documents, year of publication, name(s) of the author(s), country and institutional affiliation of each author, keywords, number of documents and their respective citations, and journal names.
Extraction format	CSV Microsoft Excel
Data processing	VOSviewer^®^ software (version 1.6.18)

**Table 2:** The 10 most cited studies between 1985 to 2023.

Authors	Study	Journal	Total Citations
[16]	Enhancement of humoral and cellular immunity by vitamin E after embryonic exposure.	*Poultry Science*	125
[17]	Effects of amino acid injection in broiler breeder eggs on embryonic growth and hatchability of chicks.	*Poultry Science*	124
[18]	Effects of in ovo injection of critical amino acids on pre- and post-hatch growth, immunocompetence, and development of digestive organs in broiler chickens.	*Asian-Australasian Journal of Animal Sciences*	77
[20]	Effects of commercial in ovo injection of carbohydrates on broiler embryogenesis.	*Poultry Science*	69
[21]	Optimum site for in ovo amino acid injection in broiler breeder eggs.	*Poultry Science*	63
[22]	Influence of different prebiotics and mode of their administration on broiler chicken performance.	*Animal*	60
[19]	Synbiotics for broiler chickens—in vitro design and evaluation of the influence on host and selected microbiota populations following in ovo delivery.	*PLoS One*	57
[23]	In ovo administration of recombinant human insulin-like growth factor-I alters postnatal growth and development of the broiler chicken.	*Poultry Science*	53
[24]	Effect of in ovo feeding of folic acid on the folate metabolism, immune function, and epigenetic modification of immune effector molecules of broiler.	*British Journal of Nutrition*	51
[25]	In ovo vaccination of specific-pathogen-free chickens with vaccines containing multiple agents.	*Avian Diseases*	51

**Table 3:** The 10 journals that published the most articles between 1985 and 2023.

Ranking	Journal	Papers	Citations	Total Link Strength	Impact Factor
1	*Poultry Science*	46	1213	144	4.014
2	*Journal of Animal Physiology and Animal Nutrition*	15	106	45	2.718
3	*Animals*	10	46	29	3.231
4	*Indian Journal of Animal Sciences*	9	84	13	0.331
5	*Iranian Journal of Applied Animal Science*	7	12	14	0.849
6	*Brazilian Journal of Poultry Science*	5	52	32	1.019
7	*Animal*	5	111	14	3.73
8	*British Poultry Science*	5	99	26	1.892
9	*Journal of Applied Poultry Research*	4	97	14	2.162
10	*Frontiers in Veterinary Science*	4	11	10	3.471

**Table 4:** Studies reviewed and respective products evaluated.

Study	Product	Effect on Hatchability *		
		Positive	Negative	Non-Existent
[31]	Amino acids			x
[32]	Ascorbic acid	x		x
	Glucose			x
[33]	L-carnitine			x
[34]	Iodinated casein + dextrin			x
[35]	L-carnitine			x
[36]	L-carnitine			x
[37]	Carbohydrate/electrolyte + potassium chloride + Theophylline			x
	Tripotassium citrate + potassium chloride + Theophylline			
	Creatine + potassium chloride + Theophylline			
[38]	L-carnitine			x
[39]	Bicarbonate		x	
	Phosphate			
	L-carnitine			
	Vitamin E			
	Vitamin C			
[40]	Vitamin C	x		x
[41]	L-carnitine		x	
[42]	25-hydroxycholecalciferol		x	x
[43]	HEPES [N-(2-hydroxyethyl)-piperazine-N′-ethanesulfonic acid]	x		
	Bicine [N,N′-Bis(2-hydroxyethyl)-glycine]	x		x
	Tris [Tris(hydroxymethyl)-aminomethane]		x	
	Bis-Tris-propane {1,3-Bis [Tris(hydroxymethyl)-methylamino] propane}	x		x
[44]	Ghrelin		x	
[28]	Royal jelly		x	x
[45]	Lysine + glutamine + glycine + proline			x
	Arginine + glutamine + glycine + proline			
	Arginine + lysine + glutamine + glycine + proline			
[46]	L-Carnitine			x
[47]	Ascorbic acid		x	
[48]	Nano form of zinc			x
	Nano form of copper			
	Nano form of selenium			
[22]	DiNovo (Extract of beta-glucans)		x	x
	Bi^2^tos (Trans-galactooligosaccharides)			x
[49]	Threonine		x	x
[50]	L-arginine		x	x
[51]	Creatine pyruvate			x
[52]	Moringa oleifera leaves	x		x
[53]	Corticotropin-releasing hormone			x
[54]	L-arginine	x		
[55]	Arginine			x
	Tryptophan			
[56]	Coccidiosis vaccination			x
[57]	Chrysin			x
	Quercetin			
	Ascorbic acid			
[58]	Lysine	x		
	Methionine			x
[59]	L-ascorbic acid			x
[60]	L-Glutamine	x		x
[61]	Zinc		x	x
[62]	VG/GA vaccine		x	x
[63]	Zinc		x	x
[64]	L-arginine		x	
	L-lysine			
	L-histidine			
[65]	Essential amino acids			x
	Linolenic acid			
	Linoleic acid			
	Retinol			
	DL-alpha-tocopherol			
[6]	Coenzyme Q10	x		
[66]	Royal jelly	x		x
[67]	Rutin		x	x
[68]	Vitamin C			x
	Vitamin B6			
	Vitamin B12			
[69]	L-ascorbic acid	x		x
[70]	*Lactobacillus animalis*			x
	*Enterococcus faecium*			
[71]	L-histidine	x		
[72]	Glucose	x		
	Egg-white protein			
[73]	L-lysine		x	
[12]	L-Arginine	x	x	x
[74]	L-glutamine + isolated soy protein	x	x	x
[75]	L-Glutamine	x	x	
[76]	Nano-selenium	x		
[13]	Canthaxanthin		x	x
[77]	Fenugreek seeds			x
	Oat seeds			
	Basil seeds		x	
[78]	Glycerol			x
	Insulin-like growth factor			
[79]	Zinc oxide nanoparticles			x
[80]	Folic acid			x
	Glucose			
[81]	Ascorbic acid	x		
	Arginine			
[82]	*Bifidobacterium bifidum*	x	x	
	*Bifidobacterium longum*			
[83]	Leucine			x
	Valine			
	Iso-leucine			
[84]	Betaine hydrochloride			x
[85]	L-Glutamine		x	
[86]	Black cumin	x		x
[87]	Clenbuterol	x		
[88]	Vitamin C	x		x
	Vitamin E			
[89]	Microalgae	x		x
[90]	Nano zinc oxide		x	
[91]	Threonine		x	x
[92]	Garlic extract	x		
	Tomato extract			
[93]	*Astragalus* polysaccharide			x
[94]	Rosemary oil	x	x	
[95]	Gaba			x
[96]	Vitamin A	x		
	Vitamin E			
	Vitamin D_3_			
	Folic acid			
[97]	Honey	x		
[98]	Essential oils (commercial blend containing star anise, cinnamon, rosemary, and thyme oil)		x	
[99]	Silver nanoparticles			x
[100]	Cysteine	x		
	Lysine			x
[101]	L-Arginine			x
[102]	Creatine monohydrate		x	
[103]	Vitamin A	x		
	L-carnitine			
	Folic acid			
[104]	L-threonine			x
[105]	Grape puree			x
	Grape pomace			
	Grape juice			
[106]	*Mycoplasma gallisepticum*			x
[107]	L-ascorbic acid			x
[108]	Garlic	x		x
[109]	Manganese		x	x
[110]	Vitamin E and ascorbic acid			x
[111]	L-arginine	x		
	L-threonine			

* Effect compared with eggs that were not subjected to any treatment; when this treatment was absent, it was compared with another control treatment.

**Table 5:** Effects of substances delivered in ovo on developing poultry including the early stages after hatching.

Study	Product	Hypothetical or Observed Effects of Substances Delivered In Ovo
[35]	L-carnitine	Stimulated embryonic metabolism and increased utilization of yolk fat and internal water content of the egg.
[42]	25-hydroxycholecalciferol	Caused a hypercalcemic condition due to an excessive influx of calcium into the circulation and tissues of the embryos.
[51]	Creatine pyruvate	Increased liver glucose, glycogen, creatine, and phosphocreatine in breast muscle and hatch weight.
[58]	Lysine	Tendency to increase relative weight.
[68]	Vitamins C, B6 and B12	Improved the immune system.
[6]	Coenzyme Q10	Improved the antioxidant status of eggs or protected against oxidative damage.
[72]	Glucose and egg white protein	Increased hatch weight.
[73]	L-lysine	Disrupted the amnion’s amino acid balance, decreased utilization of other amino acids, blocked protein synthesis, and impaired intestinal morphology.
[78]	Glycerol and insulin-like growth factor	Stimulated tissue growth, increased energy availability, amino acid supply, and intestinal villi.
[79]	Zinc oxide nanoparticles	Reduced early embryonic mortality.
[85]	L-Glutamine	Decreased yolk sac weight and increased weight of the gastrointestinal tract and pectoralis major muscle.
[76]	Nano-selenium	Improved immune response.
[90]	Nano zinc oxide	Increased early and late mortality.
[92]	Garlic and tomato extract	Increased weight and length and improved quality.
[107]	L-ascorbic acid	Increased antioxidant capacity, decreased inflammation, promoted embryonic livability, and did not impair hatching quality.
[101]	L-Arginine	Scavenged free radicals produced by physiological metabolic activities in embryonic development.
[102]	Creatine monohydrate	Increased intermediate embryonic mortality and hatch weight, stimulated the development of the heart and the total length of the gastrointestinal tract, especially important organs for digestion of nutrients (yolk sac, pro-ventricle, and gizzard), and regions for nutrient absorption (jejunum + ileum and colon + rectum).
[110]	Vitamin E and ascorbic acid	Increased yolk sac absorption.

## Data Availability

Not applicable.
